# The Serine/threonine kinase Stk33 exhibits autophosphorylation and phosphorylates the intermediate filament protein Vimentin

**DOI:** 10.1186/1471-2091-9-25

**Published:** 2008-09-23

**Authors:** Bastienne Brauksiepe, Alejandro O Mujica, Harald Herrmann, Erwin R Schmidt

**Affiliations:** 1 Institute of Molecular Genetics, Johannes Gutenberg-University, Mainz, Germany; 2 Wellcome Trust Sanger Institute, Hinxton, Cambridge, CB10 1SA, UK; 3 Division of Molecular Genetics, German Cancer Research Center, Heidelberg, Germany

## Abstract

**Background:**

Colocalization of Stk33 with vimentin by double immunofluorescence in certain cells indicated that vimentin might be a target for phosphorylation by the novel kinase Stk33. We therefore tested *in vitro *the ability of Stk33 to phosphorylate recombinant full length vimentin and amino-terminal truncated versions thereof. In order to prove that Stk33 and vimentin are also *in vivo *associated proteins co-immunoprecipitation experiments were carried out. For testing the enzymatic activity of immunoprecipitated Stk33 we incubated precipitated Stk33 with recombinant vimentin proteins. To investigate whether Stk33 binds directly to vimentin, an *in vitro *co-sedimentation assay was performed.

**Results:**

The results of the kinase assays demonstrate that Stk33 is able to specifically phosphorylate the non-α-helical amino-terminal domain of vimentin *in vitro*. Furthermore, co-immunoprecipitation experiments employing cultured cell extracts indicate that Stk33 and vimentin are associated *in vivo*. Immunoprecipitated Stk33 has enzymatic activity as shown by successful phosphorylation of recombinant vimentin proteins. The results of the co-sedimentation assay suggest that vimentin binds directly to Stk33 and that no additional protein mediates the association.

**Conclusion:**

We hypothesize that Stk33 is involved in the *in vivo *dynamics of the intermediate filament cytoskeleton by phosphorylating vimentin.

## Background

STK33/Stk33 is a serine/threonine kinase discovered in the course of sequencing the human chromosome 11 region 11p15 and mouse chromosome 7 [1]. The *Stk33 *gene in the mouse (and also *STK33 *in human) is expressed differentially in a number of specific tissues and cells like testes, lung epithelia, alveolar macrophages, and horizontal cells in the retina. In mouse embryos *Stk33 *expression is found in the developing heart, brain and spinal cord [2]. Based on sequence comparison with other kinases the STK33/Stk33 protein was classified as a member of the Ca^2+^/calmodulin-dependent kinase family (CAMK) [1,3-5].

The CAMK group is a family of multifunctional kinases: CAMK I, CAMK II and CAMK IV. Among the most well characterized CAMKs is Ca^2+^/calmodulin-dependent protein kinase II. CAMK II can phosphorylate a wide range of substrates and regulates numerous cellular functions including cell division, differentiation, cardiac contraction, and synaptic plasticity [6]. CAMK II is abundantly expressed in the brain [7] and a major effector for calcium-dependent signaling in neurons. The important neuronal function of CAMK II α has been demonstrated by analysing mice with certain mutated forms of CAMK II α [8-11]. In comparison with CAMK II α little is known about the CAMK II β subunit despite its prevailing appearance in the central nervous system. Alternative splicing variants of CAMK II β in brain with different kinase activity were identified [12]. In contrast to α and β isoforms predominantly expressed in neural tissues, the δ isoforms of CAMK II prevail in the heart [13]. The CAMK II γ isoform is mainly expressed in differentiated smooth muscle cells (dSMC). A novel variant of the γ isoform, CAMK II γ G-2, can be found in several smooth muscles, in heart and brain, but not in skeletal muscle and liver [13]. In unstimulated dSMCs it colocalizes with vimentin. Activation with a depolarizing stimulus leads to autophosphorylation of CAMK II and phosphorylation of vimentin at CAMK II specific sites. As a consequence CAMK II bound to cytoskeletal vimentin is now translocated into the cytosol. This targeting is essential for signaling in differentiated smooth muscle cells because prevention of CAMK II targeting by antisense knockdown of CAMK II γ G-2 leads to inhibition of ERK (extracellular signal-related kinase) activation as well as to inhibition of muscle contraction [13]. Anchoring CAMK II γ G-2 to vimentin in unstimulated cells is discussed as a prerequisite for optimal kinase activation or for spatial separation of the kinase and its substrate [13].

Serine 38 and serine 82 of vimentin are the major *in vitr*o and *in vivo *phosphorylation sites by CAMK II [14]. In cells infected with the cytoplasmatic DNA virus ASFV (African Swine Fever Virus) viral DNA replication resulted in activation of CAMK II and phosphorylation of vimentin on serine 82 by CAMK II. Incubation of cells with an inhibitor of CAMK II, KN93, prevented phosphorylation of vimentin and blocked both viral DNA replication and late gene expression. This underlines that CAMK activation is required for late ASFV gene expression, but the precise role played by CAMK II in ASFV DNA replication is still unknown. In virus infected cells vimentin phosphorylated on serine 82 disassembles into aggregates which are transported along microtubules and are reorganized into a cage like structure around virus assembly site. This vimentin cage has on the one hand a cytoprotective function by preventing the diffusion of viral components into cytoplasm and on the other hand it concentrates late structural proteins at site of virus assembly [15].

The phosphorylation/dephosphorylation state regulates the dynamic behaviour of the intermediate filament cytoskeleton. The major vimentin phosphatase *in vivo *is type 1 protein phosphatase (PP1) [16]. PP1c is *in vivo *associated with vimentin and dephosphorylates the CAMK II-specific phosphorylation sites of vimentin Ser38 and Ser82. Phospho-Ser82 of vimentin is dephosphorylated much slower than phospho-Ser38 by PP1c [17]. This delayed Ser82 dephosphorylation might influence the dynamics of vimentin filament assembly/disassembly. A requirement for cell division during mitosis is the reorganization of the intermediate filament system through phosphorylation of vimentin as demonstrated by site-specific mutation of vimentin [18]. In the case of Polo-like kinase 1 (Plk1), a kinase which also phosphorylates vimentin Ser82 [19], elevated Ser82 phosphorylation by Plk1 may play a role in efficient segregation of vimentin filaments during mitosis [19] as phospho-Ser82 on vimentin is hardly dephosphorylated by PP1 in mitosis. Phospho-Ser82 may act as a memory phosphorylation site [17].

The different sites available for serine/threonine phosphorylation in vimentin are targeted by different kinases [18]. Since we found the striking colocalization of vimentin and Stk33 in various cell types and tissues (manuscript in preparation), we were prompted to investigate whether Stk33 might be another kinase to phosphorylate vimentin.

Stk33 is a serine/threonine kinase of so far unknown function. In the present study we used bacterially expressed recombinant mouse Stk33 with several artificial vimentin deletion mutant polypeptides also expressed as recombinant proteins in *E. coli *for *in vitro *phosphorylation assays. In addition we performed co-immunoprecipitation experiments with protein extracts obtained from the mouse Sertoli cell culture SerW3. We know from previous studies (manuscript in preparation) that Stk33 and vimentin are coexpressed and colocalized in Sertoli cells of mouse testes. So, Sertoli cells should be an ideal resource for gaining native interacting Stk33 and vimentin.

However, from our previous studies it is also clear, that Stk33 is not always expressed in cells together with vimentin. We find Stk33 in vimentin-negative cells, too.

The differential expression pattern of Stk33 in mice and men resembles those of some members of the CAMK-Group [2]. Stk33 and CAMKII might have similar functions for example in the dynamic regulation of the intermediate filament system by phosphorylation in the course of the separation of daughter cells during mitosis. Stk33 is expressed very specifically in some organs of the developing mouse embryo [2]. Thus, Stk33 could play also a role in organ development in addition to its function in phosphorylating vimentin. In this study, however we show that vimentin is a target for phosphorylation by Stk33 *in vitro *and that Stk33 and vimentin can be co-immunoprecipitated indicating a close interaction also *in vivo*.

## Results

### Kinase assay

**Figure 1:**
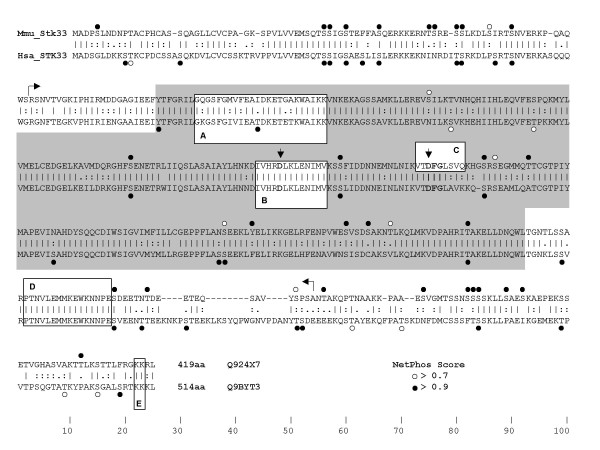
**Aligment of mouse-human Stk33/STK33 with relevant kinase features indicated.** Potential phosphorylation sites predicted using the NetPhos 2.0 Server are shown as dots over serine, threonine and tyrosine residues. NetPhos prediction compares all strings of +/- 4 aa around each S/T/Y along the sequence with known experimentally obtained phosphorylation sites [46]. Net Phos default cut off value used is 0.5. To increase the confidence of the predictions, only those equal or higher than 0.7 are shown and sites scoring 0.9 and above are shown with full circles. Horizontal arrows mark the N- and C-terminus of the recombinant Stk33 protein fragment. The grey box highlights the protein kinase domain following Hanks and Hunter canonical description [20]. **A**: Protein kinases ATP-binding region signature (Prosite PS00107). **B**: Serine/threonine protein kinases active-site signature (Prosite PS00108). Additionally a vertical arrow points at the consensus aspartate residue recognized in the active site. **C**: Deleted amino acids in Stk33δ. Vertical arrow shows aspartate residue in the consensus DFG, the phosphate donor ATP anchoring site. **D**: Peptide sequence targeted by the antibody used in this work. **E**: Conserved di-lysine C-terminal motif that might be involved in ER anchoring [47].

**Figure 2:**
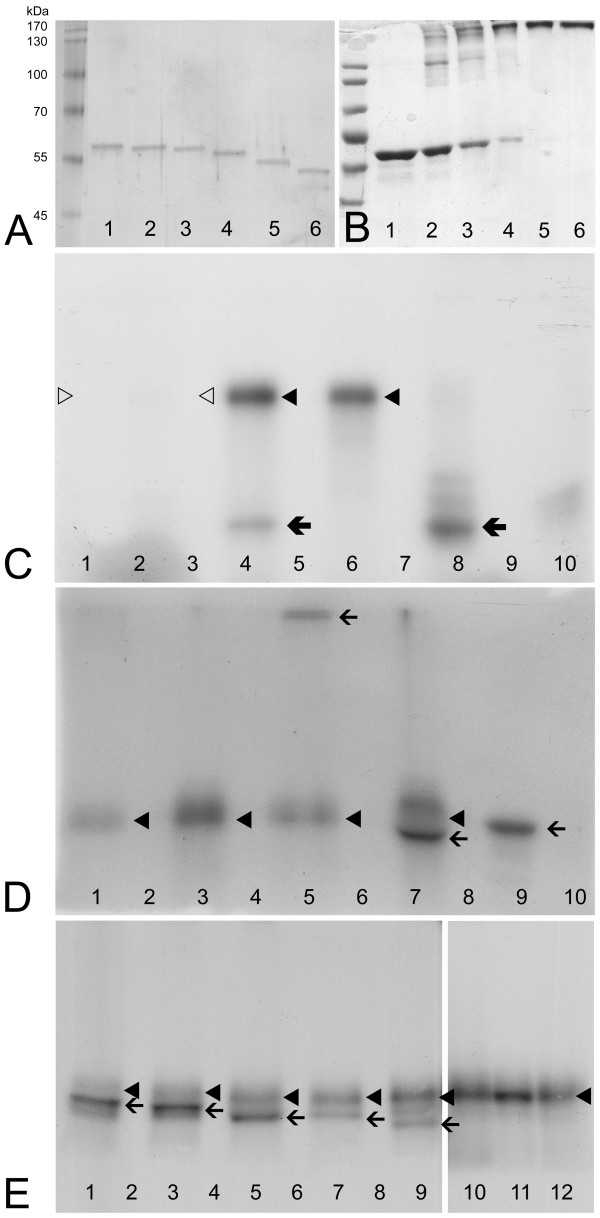
**Phosphorylation assay**. **A**: Silver stained gel after electrophoretic separation of wildtype (wt) vimentin and different deletion variants of vimentin used in the kinase assay as substrates. Lane 1: wildtype, lane 2: Δ12, lane 3: Δ20, lane 4: Δ30, lane 5: Δ42, lane 6: Δ50.**B**: Coomassie stained gel of crosslinked vimentin wildtype monomers by using increasing concentration of glutaraldehyde (GA). For practical reasons (see text) crosslinked vimentin tetramers had to be used in the kinase assay. Lane 1: vim wt without GA, lane 2: vim wt plus 0.005% GA, lane 3: vim wt plus 0.01% GA, lane 4: vim wt plus 0.02% GA, lane 5: vim wt plus 0.04% GA, lane 6: vim wt plus 0.06% GA. **C**: Electrophoretic separation of the products of different kinase assay with various reactions partners after *in vitro *incubation with radiolabeled γ ^32^P ATP and autoradiography of the gel. Lane 1: Only Stk33δ (deletion derivative) tested for autophosphorylation, lane 2: Stk33δ plus casein as substrate, lane 3: Stk33δ plus vimentin wildtype, lane 4: Stk33 (complete kinase domain) plus casein, lane 6: only Stk33 tested for autophosphorylation, lane 8: Protein kinase A (PKA) plus casein as substrate, lane 9: only PKA tested for autophosphorylation, lane 10: casein + γ ^32^P ATP only. Lanes 5 and 7 are devoid of samples. **D**: Electrophoretic separation of Stk33 and vimentin/vimentin deletion derivatives after *in vitro *incubation with radiolabeled γ ^32^P ATP and autoradiography of the gel. Lane 1: Stk33 plus ΔH crosslinked, lane 3: Stk33 autophosphorylation, lane 5: Stk33 plus vimentin wildtype tetramer, lane 6: vimentin wildtype tetramer plus γ ^32^P ATP only, lane 7: Stk33 plus vimentin monomer, lane 9: PKA plus vimentin monomer, lane 10: PKA autophosphorylation. Lanes 2, 4 and 8 are devoid of samples. **E**: Electrophoretic separation of Stk33 and vimentin/vimentin deletion derivatives after *in vitro *incubation with radiolabeled γ ^32^P ATP and autoradiography of the gel. Lane 1: Stk33 plus vim Δ12, lane 3: Stk33 plus vim Δ20, lane 5: Stk33 plus vim Δ30, lane 7: Stk33 plus vim Δ42, lane 9: Stk33 plus vim Δ50, lane 10: Stk33 plus vim ΔH, lane 11: Stk33 plus vim wt, lane 12: Stk33 autophosphorylation. Lanes 2, 4, 6 and 8 are devoid of samples. Thin arrows indicate vimentin/vimentin deletion derivatives as substrate, thick black arrows indicate casein as substrate, black arrowheads indicate Stk33, white arrowheads indicate Stk33δ. To assure the results presented all experiments were carried out at least two times. Some of the assays were iterated up to four times as a positive control.

**Figure 3:**
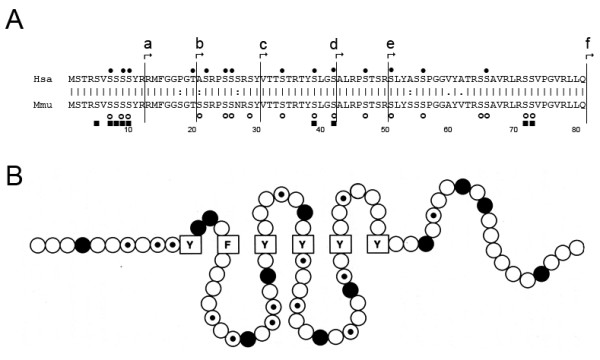
**A**: **Amino acid sequence alignment of the non helical head domain (H) of vimentin from human (Hsa) and mouse (Mmu).** Starting points of the truncated vimentin derivatives used in this study are indicated (a = Δ12, b = Δ 20, c = Δ 30, d = Δ 42, e = Δ 50, f = Δ H). The numbers indicate how many amino acids were deleted from the amino-terminus. Phosphorylation sites in the vimentin head domain are indicated as reviewed in [33]. Black dots represent phosphorylation sites on human vimentin as mentioned in [26,35]. Open circles indicate phosphorylation sites in the mouse vimentin head domain according to [34]. Vimentin phosphorylation sites found in the hamster are symbolized by black squares [21]. **B**: A hypothetical structural model for the human vimentin head domain. Amino acids are represented by circles or boxes, aromatic amino acids are boxed, basic ones are filled, and potential phosphorylation sites are dotted. Figure modified from [26].

The recombinant Stk33 enzyme was expressed in *E. coli *and affinity purified [2]. It contains all canonical kinase subdomains and signatures [20] and also the epitope for the anti-Stk33 antibody used in this study, which is located N-terminal to the kinase domain (Figure 1). In addition to nearly full length enzyme, a naturally occurring splice variant of Stk33, Stk33δ was also tested in the assay. This splice variant was cloned and expressed as described for recombinant nearly full length Stk33 [2] [GenBank:AM056057]. In this splice variant, there are 27 base pairs within the kinase domain missing. Parts of the missing amino acid sequence include the DFG-triplet [20] (Figure 1C). It is known, these amino acids are responsible for anchoring the phosphate donor ATP [20]. So this splice variant should produce an inactive form of Stk33 kinase. The recombinantly expressed Stk33δ isoform was tested for both autophosphorylation and phosphorylation of substrates such as vimentin wildtype and casein. As a positive control PKA catalytic subunit was used because it readily phosphorylates casein. This PKA catalytic subunit is not able to phosphorylate itself. Recombinant vimentin wildtype protein and different deletion derivatives expressed in *E. coli *were used as major targets in the assay. One technical problem had to be circumvented: Recombinant Stk33 and wildtype vimentin have a very similar electrophoretic mobility, and as we could show Stk33 is able to perform autophosphorylation. Thus on normal PAGE a discrimination between autophosphorylated Stk33 and phosphorylated wildtype vimentin is hardly possible. Therefore, the vimentin wildtype monomer was treated with increasing concentrations of glutaraldehyde [21] to form crosslinked vimentin tetramers with an apparent molecular weight of approximately 180 kDa (Figure 2B). This tetramer form of vimentin was included in the phosphorylation assay. Furthermore a number of vimentin mutants with different molecular weights were used in the kinase assay (Figure 2A, lane 2–6 and Figure 3). The deletion variants of human vimentin used are Δ12, Δ 20, Δ 30, Δ 42 and Δ 50, the numbers indicate how many amino acids were deleted from the amino-terminus (Figure 3). In addition, a mutant vimentin missing the entire non-α-helical amino-terminal domain ("head") was employed.

Recombinant mouse Stk33 kinase was incubated with γ ^32^P ATP with the various substrates under optimized conditions tested previously. The reaction products were separated by SDS-PAGE and detected by direct autoradiography. The results clearly demonstrate (autoradiographs shown in Figure 2C, D, E) that: i) Stk33 (complete kinase domain) is able to perform autophosphorylation. The incubation of Stk33 without any other substrate (lane Stk33 only; Figure 2C, lane 6; Figure 2D, lanes 3, Figure 2E, lane 12) leads to a strong radiolabeled band with an apparent molecular weight corresponding to the one of Stk33 (black arrowhead); ii) The derivative of Stk33, Stk33δ in which part of the kinase domain is deleted is not able to perform autophosphorylation (Figure 2C, lanes 1–3; white arrowhead). Furthermore, Stk33δ is not able to phosphorylate any of the tested substrates (Figure 2C, lane 2 and 3) in contrast to Stk33 (Figure 2C, lane 4); iii) Stk33 clearly phosphorylates vimentin *in vitro *(Figure 2D, lane 5 and 7; Figure 2E, lanes 1, 3, 5, 7, 9; arrows). By using the vimentin tetramer as substrate, differentiation between autophosphorylated Stk33 and phosphorylated vimentin wildtype is clearly possible (Figure 2D, lane 5; arrow).

When Stk33 is incubated together with wildtpye vimentin and the deletion variants Δ12 to Δ50, a preferred phosphorylation of vimentin over Stk33 is observed, however, there is always a basic autophosphorylation of Stk33 recognizable (black arrowhead in Figure 2E, lanes 1, 3, 5, 7, 9). So far it is not clear, whether the autophosphorylation is a prerequisite for the kinasing activity of Stk33 or whether also unphosphorylated Stk33 is able to phosphorylate vimentin. The limited resolution of deletion variants Δ30 and Δ42 in the gel might be related to a different extent of phosphorylation in truncated vimentin Δ30 compared to Δ42 and therefore a changed electrophoretic mobility might be the consequence, but this is speculation.

Notably, the truncation mutants Δ12, Δ20 and Δ30 are phosphorylated to a higher extent than Δ42 and Δ50 (Figure 2E). Therefore we conclude that, since headless vimentin is not phosphorylated at all, only the head domain of vimentin is phosphorylated and furthermore that sites both on the first 30 amino acids and sites after amino acid 30 up to the end of the head domain are phosphorylated.

As seen in Figure 2E, lane 11 it is difficult to visualize the only minute different position between autophosphorylated Stk33 and vimentin on an autoradiograph as Stk33 and vimentin have the same electrophoretic migration behaviour in a PAGE. The samples on the gel of the autoradiography 2 D were electrophoretically resolved by a longer running time than 2 E to achieve a better separation. Therefore, in lane 11, Figure 2E (shorter running time) the resolution is not good enough to resolve the two proteins of nearly identical molecular weight and electrophoretic mobility. Vimentin phosporylated by Stk33 (lane 7, Figure 2D) or by PKA (lane 9, Figure 2D) appears to migrate slightly different. The extent of phosphorylation might be different inducing a phosphorylation-dependent mobility shift on gels.

### Immunoprecipitation

In order to prove that Stk33 and vimentin are also *in vivo *associated proteins co-immunoprecipitation experiments were carried out. As a positive control recombinant nearly full length Stk33 protein was precipitated using protein A sepharose and anti-Stk33 antibody [2]. For all co-immunoprecipitation experiments Sertoli cell culture SerW3 was used. All protein samples (total protein extract from SerW3, samples of washing steps, precipitated proteins and recombinant proteins as positive controls for Western detection) were analyzed by SDS-PAGE after preheating in non-reducing Laemmli buffer. Therefore, the main portion of IgG molecules is still present. The appearance of a band corresponding to the IgG heavy chain is perhaps explainable because of heating during probe preparation.

**Figure 4:**
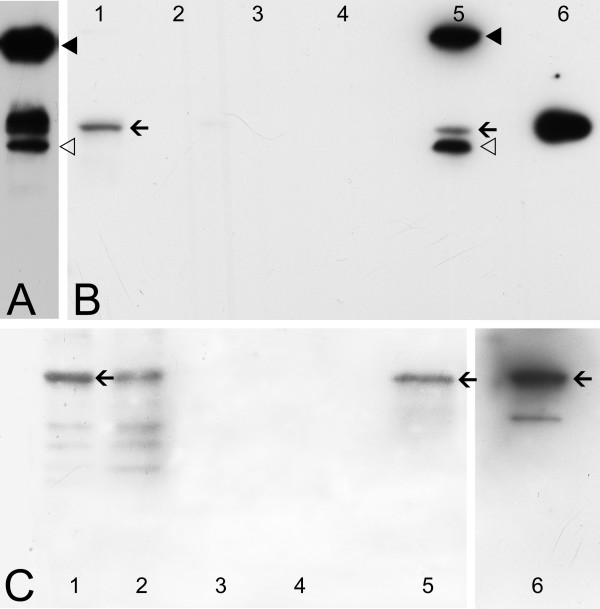
**Immunoblotting analysis of co-immunoprecipitation assays using anti-Stk33 (A and B) and anti-vimentin (C) for detection**. **A**: Western analysis of immunoprecipitated recombinant Stk33 with anti-Stk33 as positive control (arrowheads point towards IgG contamination). **B**: Western analysis using anti-Stk33 antibody for detection. Immunoprecipitation was carried out using anti-Stk33 and SerW3 cultured cell extracts; lane 1: protein extract from SerW3 cell culture; lanes 2, 3, 4: samples of washing step 1, step 2, and step 3; lane 5: immunoprecipitate; lane 6: recombinant Stk33. Arrows = Stk33, arrowheads = IgG. **C**: Western analysis using anti-vimentin antibody for detection. Co-immunoprecipitation of vimentin was carried out with anti-Stk33. Lane 1: protein extract from SerW3 cell culture; lanes 2, 3, 4: samples of washing step 1, step 2, step 3; lane 5: immunoprecipitate; lane 6: recombinant vimentin. Arrows = vimentin. The results presented were repeated twice.

Cellular Stk33 could be readily immunoprecipitated from protein extracts of cultured Sertoli cells SerW3 (Figure 4B, lane 5; arrow). The apparent molecular weight of the immunoprecipitated Stk33 is obviously the same as the Stk33 protein detected in unprecipitated SerW3 total protein extracts (Figure 4B, lane 1; arrow). The additional bands (very strong signals, filled and open arrowheads) probably arise from the IgG molecules not removed from the reactions mixtures (upper band, filled arrowhead – whole IgG; lower band, open arrowhead – IgG heavy chain).

To analyse whether vimentin was co-precipitated by the precipitation with anti-Stk33, a Western Blot analysis was carried out with the identical material as used for the Western blot in Figure 4B but for the detection an anti-vimentin antibody was used. Recombinant human vimentin wildtype protein was included in the analysis as a positive control (Figure 4C, lane 6). Co-immunoprecipitation from cellular extracts suggests that Stk33 and vimentin could be associated *in vivo *(Figure 4C, lane 5; arrow). In addition to the detection of vimentin in the precipitate, there was some vimentin in the washing buffer of the first washing step (Figure 4C, lane 2). The following washing steps did not show any vimentin in the washing buffer (Figure 4C, lane 3 and 4), and hence it is highly improbable that insufficient washing of the precipitate is responsible for the vimentin content. The results indicate clearly, that Stk33 and vimentin are *in vivo *associated proteins.

### Co-sedimentation assay

**Figure 5:**
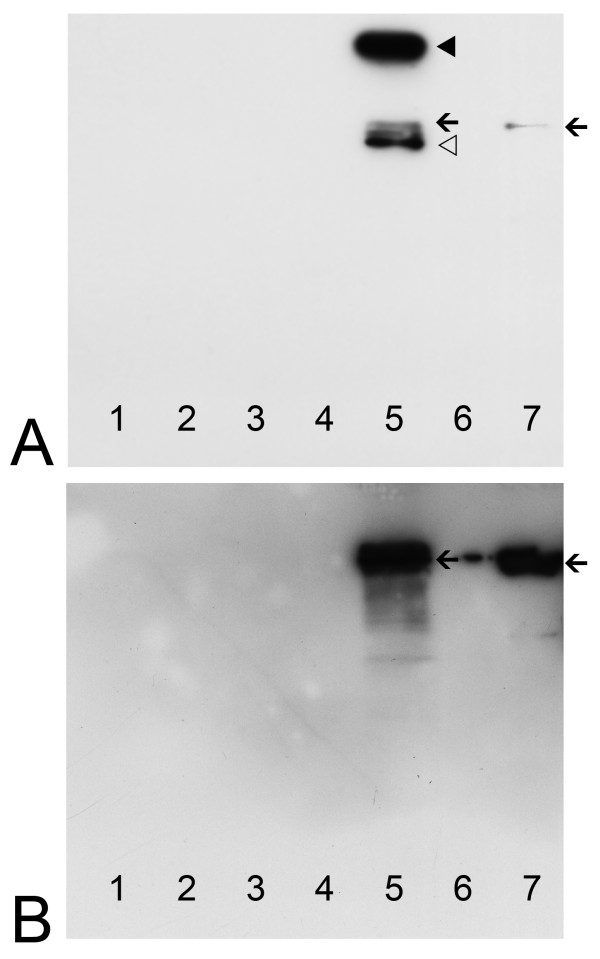
**Co-sedimentation assay of recombinant Stk33 and recombinant vimentin ΔN50 using anti-Stk33 for precipitation**. **A**: Immunoblotting analysis of sedimentated proteins using anti-Stk33 for detection. Lane 1–3: samples of washing steps 1–3; lane 5: co-sedimentation sample; lane 7: recombinant Stk33; lane 4 and 6 were free of sample. The arrowheads point towards IgG contamination. Arrow = Stk33. **B**: Immunoblotting analysis of sedimentated proteins using anti-vimentin for detection. Lane 1–3: samples of washing steps 1–3; lane 5: co-sedimentation sample; lane 7: recombinant vimentin ΔN50; lane 4 and 6 were free of sample. The protein detected in lane 6 is due to protein of lane 7 spilled over. As expected no IgG contamination is visible since an anti-mouse IgG peroxidase conjugate was used for detection of anti-vimentin and utilized anti-Stk33 is produced in rabbit. This assay was confirmed twice.

To test whether Stk33 binds directly to vimentin, an *in vitro *co-sedimentation assay was performed. By analyzing the sedimented proteins via Western Blotting experiments we could show, that recombinant Stk33 precipitated (Figure 5A, lane 5) and that vimentin is successfully co-sedimentated (Figure 5B, lane 5) using anti-Stk33 for precipitation. This suggests that vimentin binds directly to Stk33 and that none intermediate protein mediates the association.

### Testing enzymatic activity of immunoprecipitated Stk33 by a kinase assay

**Figure 6:**
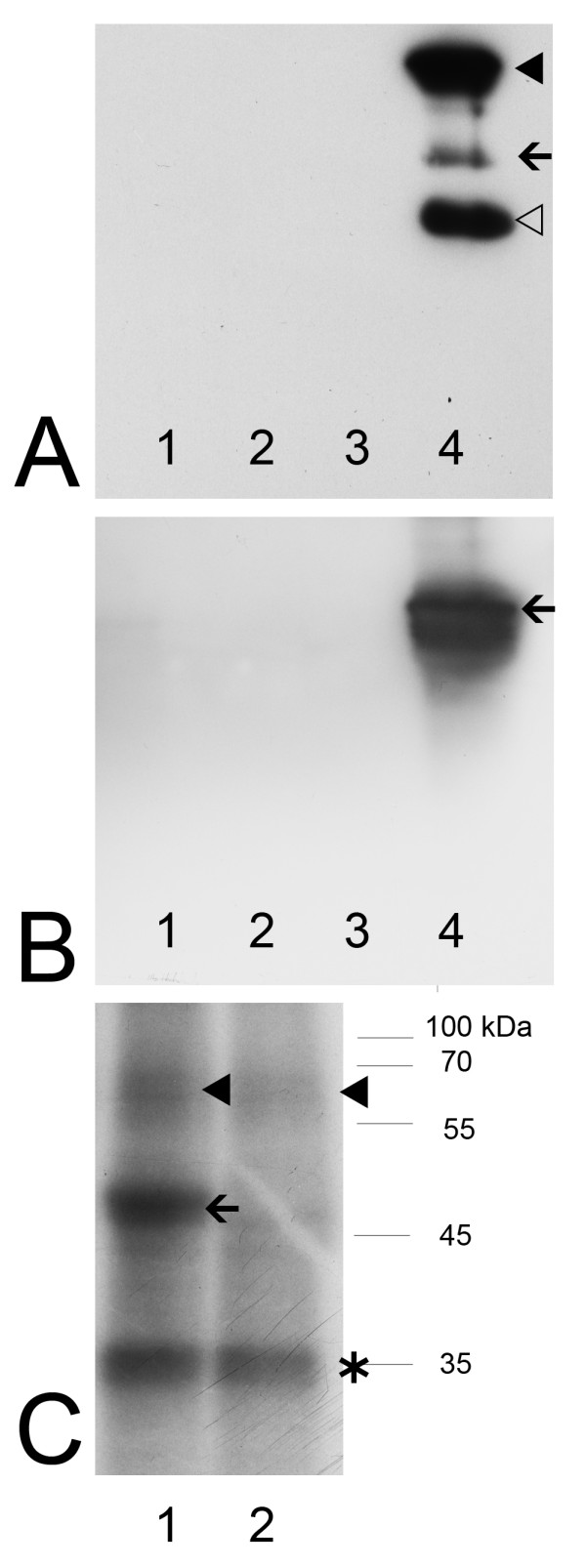
**Analysis of a co-immunoprecipitation assay by immunoblotting (A and B) and by phosphorylation assay (C)**. **A**: Western Blot analysis using anti-Stk33 for the detection of precipitated Stk33. Lane 1–3: samples of washing steps 1–3; lane 4: immunoprecipitate; the arrowheads point towards IgG contamination. Arrow = Stk33. **B**: Western Blot analysis using anti-vimentin for the detection of co-precipitated vimentin. Lane 1–3: samples of washing steps 1–3; lane 4: immunoprecipitate; Arrow = co-precipitated vimentin. **C**: Phosphorylation assay proofing the enzymatic activity of immunoprecipitated Stk33. Electrophoretic separation of the precipitate after *in vitro *incubation with radiolabeled γ ^32^P ATP and autoradiography of the gel. An aliquot of the precipitate was incubated with recombinant vimentin ΔN50 (lane 1) and with recombinant vimentin ΔH (lane 2). Arrowheads indicate phosphorylated Stk33 and/or vimentin as a discrimination is not possible due to similar molecular weight of the proteins. The radioactively labelled protein band (arrow) corresponds to phosphorylated vimentin ΔN50 according to co-electrophoresed molecular standard. * indicates an additional protein that co-precipitated by anti-Stk33. The phosphorylation assay was done two times.

The incubation of radioactive γ ^32^P ATP and recombinant vimentin protein with the co-immunocomplex obtained by co-precipitation of Stk33 and vimentin shows that immunoprecipitated Stk33 has kinase activity (Figure 6C, arrowhead) and is able to phosphorylate recombinant vimentin (Figure 6C, arrow). The enzymatic activity of the precipitated enzyme might be reduced by storing the immunocomplex at 4°C during time for Western Blotting experiments to check for successful co-immunoprecipitation which is a prerequirement for testing the enzymatic activity of Stk33. A third protein with an apparent molecular weight of about 35 kDa seems to be also co-precipitated by anti-Stk33 since a radioactively labelled additional band appears after autoradiography (Figure 6C, asterisk). As this additional band is not visible after anti-Stk33 reaction it is probably not a splice variant of Stk33.

## Discussion

The aim of the present study was to investigate whether the novel serine/threonine kinase Stk33 phosporylates the intermediate filament protein vimentin. The motivation to test this was a striking colocalization of vimentin and Stk33 in various tissues and differentiated cells (manuscript in preparation). The results of the *in vitro *kinase assays and of the co-immunoprecipitation studies are very clear:

Stk33 is able to phosporylate vimentin *in vitro *and vimentin and Stk33 form a complex *in vivo *which can be readily co-precipitated by the use of an anti-Stk33 antibody. Stk33 binds directly to vimentin as determined by the co-sedimentation assay. Therefore, none intermediate protein mediates this association. We conclude that Stk33 plays a specific role in the dynamic behaviour of the intermediate filament cytoskeleton by phosphorylation of vimentin. It is known that phylogenetically related genes often have similar functions. Thus it is not too surprising that Stk33 -a member of the family of Ca^2+^/calmodulin-dependent protein kinases [1]- is able to phosphorylate vimentin. CAMKII among other kinases is one of the major kinases responsible for the phosphorylation of the cytoskeletal protein vimentin [22]. In that respect it is interesting that Stk33 undergoes autophosphorylation. Whether the autophosphorylation of Stk33 is a prerequisite for the phosphorylation of vimentin is not known. However, it is known, that autophosphorylation is the key event in the phosphorylation process of other members of the CAMK group [23,24].

Intermediate filament proteins form the largest family of cytoskeletal proteins in mammalian cells. Intermediate filament proteins can be classificated into six types based on their gene structure, sequence homology and immunological and/or assembly properties [25]. Vimentin belongs to type III of intermediate filament proteins which also comprises desmin, GFAP and peripherin.

IF proteins are composed of an amino-terminal head, a central rod and a carboxy-terminal tail [26,27]. The rod domain is subdivided into further segments by non-α-helical regions, called linker. The head domain is essential for IF assembly and the tail for the control of lateral association. Dimerization is mediated by the rod-domain.

Most of the kinases phosphorylate sites on IF proteins located in the amino-terminal non α-helical head domain (e.g. cdc2 kinase [28], cAMP-dependent protein kinase (protein kinase A) [29], protein kinase C [29], CaMKII [22], p21-activated kinase (PAK) [30-32]). Stk33 shows similar head domain specificity: It phosphorylates different head domain deletion derivatives, but is not able to phosphorylate vimentin in which the head domain is deleted completely. Vimentin with 50 amino acids missing from the animo-terminus is still phosphorylated by Stk33 in contrast to vimentin missing 80 amino acids. Therefore we conclude that Stk33 phosphorylates one or more of the phosphorylation sites known from other kinases. The potential phosphorylation sites beyond the head domain (downstream of amino acid 81) [33,34] and in the tail domain of vimentin [21,33,35] are not phosphorylated by Stk33. We can therefore be rather confident that the phosphorylation sites for Stk33 are located in the vimentin head-domain.

In spite of the differentiation- and tissue-specific expression patterns, the function of the intermediate filament proteins has long been considered to be just structural. By forming a continuous network stretched from the nuclear surface to the cell membrane and associated in tight interaction with the nuclear lamina and the nuclear cytoskeleton, it is assumed that intermediate filaments modulate and control signal transduction [25]. The dynamic behaviour of the intermediate filament cytoskeleton is under control of kinases and phosphatases leading to structural changes of the intermediate filament cytoskeleton like reorganization, solubilization or collapse. Various types of serine/threonine protein kinases phosphorylate intermediate filament proteins *in vitro *leading to disassembly of the filament structure [14]. Up to now we do not yet know whether phosphorylation of vimentin by Stk33 causes disassembly albeit this is conceivable because high levels of vimentin phosphorylation often lead to structural alterations of the filament system.

The remodelling between polymerized intermediate filaments (long filaments and short filaments called squiggles) and non-filamentous particles is regulated by kinases [36]. Among the different structural filament forms the non-filamentous precursors (particles) are the most interesting [37]. It has been reported that these particles can move long distances at high speed along microtubules with the help of molecular motors [38-40]. Filament precursors are delivered to special regions within the cell, where an assembly to long intermediate filaments takes place. Such flexibility enables cell movement and reorganization of the cytoplasm. Interestingly, the bi-directional movement of vimentin intermediate filaments along microtubule enables a kinase signaling over long distances within a cell. This is of special interest in neurons, where signals generated in axonal or dendritic processes have to travel long distances to the cell body (retrograde transport), especially to the nucleus, where the kinase can affect gene expression. The transport complex consisting of phosphorylated MAP kinase Erk1/2, importin β and dynein requires vimentin particles for movement along microtubule fibres in injured neurons [41]. Normally, adult neurons express only terminally differentiated neuronal intermediate filament proteins like neurofilament proteins, but translationally silenced vimentin mRNA is activated and synthesized in lesioned nerves. As only soluble vimentin particles are capable of binding a kinase, de novo synthesized vimentin protein has to be disassembled into particles by phosphorylation or by modification through proteolysis [37]. De novo synthesized vimentin is therefore exposed to high calcium concentrations that prevent assembly of vimentin particles to filaments due to vimentin phosphorylation by CAMKII [42] or due to calpain-mediated cleavage of vimentin [41]. The creation of the kinase/vimentin complex is only possible with a phosphorylated kinase and it is promoted at high Ca^2+^concentrations (e.g. near site of nerve injury). On the contrary, the complex dissociates near the cell body where the Ca^2+ ^concentrations are low [41]. During the retrograde transport dephosphorylation of pErk is avoided as long as vimentin stays bound to the kinase. Vimentin hides phosphorylated residues in the kinase and therefore confines the access of the phosphatase to these residues [37]. Furthermore, other interacting partners are not capable of binding to the kinase, which in turn guarantees the specificity of the transmitted signal. Interestingly, this kinase transport mechanism described for lesioned nerves is not possible in vimentin null mice [41]. Besides several defects (e.g. in cerebellar glia [43]) vimentin-null mice show a defective wound repair [44], which might be related to the deficit of vimentin-dependent signaling as described for lesioned nerves [37].

The dynamic changes of the intermediate filament organization are particularly prominent during cell movement or mitosis and cell division. There is a constant state of flux between non-filamentous components, short filaments and long filaments. In some but not all cell types, vimentin filaments disassemble into aggregates and short filaments during metaphase [45]. The organizational changes observed during mitosis are accompanied by a significant increase in the phosphorylation state. Site-specific mutation of vimentin and therefore changes in potential phosphorylation sites have been demonstrated to induce the formation of intermediate filament bridges between unseparated daughter cells [18]. To elucidate the precise molecular function of Stk33 in vimentin phosphorylation, it is important to determine the specific phosphorylation sites on vimentin by Stk33 which is planned for the future.

## Conclusion

Our results show that the serine/threonine kinase Stk33 phosphorylates the intermediate filament protein vimentin *in vitro *specifically in the vimentin head domain. Stk33 undergoes obligatory autophosphorylation, which might be a prerequisite for its kinasing activity. By co-immunoprecipitation we were able to co-isolate vimentin together with Stk33 using a polyclonal anti-Stk33 antibody. From this result we conclude that Stk33 and vimentin are interacting protein partners also *in vivo*. This conclusion is strongly supported by the observation that Stk33 and vimentin can be found together in many very specialized cells and tissues (manuscript in preparation). We propose that Stk33 is involved in the dynamics of intermediate filament assembly/disassembly through a specific and regulated phosphorylation of vimentin.

## Methods

### Kinase assay

The kinasing activity of Stk33 was determined in an *in vitro *kinase assay. 0.32 μg recombinant Stk33 and 1.75 μg vimentin/vimentin deletion derivatives were incubated with 10 mM MgCl_2 _in 1× kinase buffer (Na-Hepes pH 7.0, 0.05% Briji). In order to use crosslinked vimentin tetramers as a substrate for Stk33 increasing concentrations of glutaraldehyde (0; 0.005; 0.01; 0.02; 0.04; 0.06%) [21] were used to form these complexes. As a substrate positive control casein phosphorylated by both Stk33 and Protein kinase A (PKA) catalytic subunit (Sigma) was applied to the assay. As a control for a contamination with any endogenous kinase, negative controls (assay without additionally applied Stk33 or PKA) were carried out. The reaction was initiated by adding 20 μCi γ ^32^P ATP. After incubation for 2 hours at 30°C, the reaction was stopped by adding SDS-sample buffer (125 mM Tris, 4% SDS, 20% Glycerol, 10 mM β-Mercaptoethanol, 2 mM EDTA, 0.04% Bromphenol blue, pH6.8). Samples were boiled for 5 min prior to loading onto polyacrylamide gels and separation by SDS-PAGE. Gels were finally autoradiographed by exposure to Kodak X-AR films.

### Co-immunoprecipitation and Western Blotting

For immunoprecipitation, SerW3 cells (kindly provided by Prof. Dr. Oesch, University Hospital Mainz) were washed twice with PBS, scraped off, and solubilized in ice-cold lysis buffer containing 1% NP-40, 5 mM EDTA, 2 mM PMSF in PBS (pH 7.4) by incubation at 4°C in a shaker for 1 h. Lysed cells were centrifugated to remove particles for 20 minutes with 14 000 rpm at 4°C. Anti-Stk33 [2] was added to the supernatant. After incubation for 2 h, protein A sepharose (Amersham Bioscience Europe GmbH, Freiburg) was added for 1 h under constant agitation. After a centrifugation step (500 rpm, 4°C, 30 seconds) the pellet was washed three times with 1 ml washing buffer (0.1% NP-40, 5 mM EDTA in PBS, pH 7.4). The final pellet was suspended in non-reducing Laemmli buffer, heated to 95°C for 3 minutes and subjected to SDS-PAGE. Western Blotting experiments were carried out as described previously [2] using PVDF-membrane (Roth) and Immobilion Western-HRP chemiluminescence substrate (Millipore). For the detection of immunoprecipitated proteins, a polyconal anti-vimentin antibody kindly provided by Prof. Leube, University of Mainz, Germany was applied (1:15000). Anti-guinea pig-HRP as secondary antibody was used at 1:24000 dilution in PBS-T.

### Co-sedimentation assay

For an *in vitro *co-sedimentation assay, recombinant Stk33 and vimentin ΔN50 protein were incubated in phosphate buffered saline for 2 h at 4°C under gentle agitation. Recombinant Stk33 was precipitated from the solution by using anti-Stk33 antibody and protein A sepharose (Amersham Bioscience Europe GmbH, Freiburg). After centrifugation the sedimented material was washed 3 times with 1 ml washing buffer (0.1% Nonidet P40, 5 mM EDTA). Aliquots of all washing samples and the final pellet were suspended in non-reducing buffer, heated and electrophoretically resolved by gel electrophoresis. To test whether Stk33 and vimentin sedimented together, Western Blotting experiments were carried out using anti-Stk33 and monoclonal anti-rabbit IgG peroxidase conjugate clone RG-96 (Sigma) and anti-mouse IgG peroxidase conjugate (Sigma) for the detection of anti-vimentin Ab-2 (Dianova).

### Testing enzymatic activity of immunoprecipitated Stk33 by kinase assay

In order to test whether the immunoprecipitated Stk33 has enzymatic activity we incubated precipitated Stk33 with recombinant vimentin proteins. Co-immunoprecipitation was carried out as described before. As a discrimination between autophosphorylated Stk33 and phosphorylated vimentin is hardly possible we used recombinant vimentin with a deletion of 50 amino acids for the phosphorylation studies. The reaction was initiated by adding 20 μCi γ ^32^P ATP. After 1 hour at 30°C the reaction was stopped by adding non-reducing Laemmli buffer. Further steps were carried out as already described in the kinase assay protocol.

## Authors' contributions

BB had the initial idea that vimentin might be a substrate for the Stk33 kinase and planned and carried out all experiments. BB wrote also the first draft of the manuscript and prepared the figures. AM was involved in designing the kinase assays and he initially discovered the Stk33 gene and provided the sequence and the DNA for Stk33. HH provided the essential recombinant vimentin protein and deletion derivatives thereof. ERS started as principle investigator the entire research programme in which course the Stk33 gene was discovered, initiated the functional analysis of Stk33, contributed substantially to various versions including the final version of the manuscript and has given final approval of the version to be published. All authors read and approved the final manuscript.
